# Neuroprotective Effect of Cannabidiol Against Rotenone in Hippocampal Neuron Culture

**DOI:** 10.1002/asia.202500946

**Published:** 2025-11-19

**Authors:** Seoin Yang, Hyunwoo Choi, Jungnam Kim, Insung S. Choi

**Affiliations:** ^1^ Department of Chemistry KAIST Daejeon Republic of Korea

**Keywords:** cannabidiol, neuroprotection, primary hippocampal neurons, rotenone, TRPV1

## Abstract

(–)‐Cannabidiol (CBD), a non‐psychoactive phytocannabinoid, has been suggested to provide protective effects in neuronal systems. This work investigates its neuroprotective effect against rotenone, a mitochondrial complex I inhibitor that causes neuronal toxicity, using primary hippocampal neurons. Rotenone treatment reduces neuronal viability with marked neurite degeneration in a concentration‐dependent manner (LC_50_ = 189.1 nM). Administration of 2.5 µM CBD significantly increases viability to 69.9%, compared with 45.6% observed under 200 nM rotenone treatment. Neuronal morphology is preserved under both CBD pre‐treatment and co‐treatment conditions, with confocal analyses further confirming the maintenance of axonal branching and overall structural integrity. Antagonist experiments reveal that TRPV1 inhibition markedly reduces the protective effect of CBD, whereas blockade of 5‐HT_1A_R has only a minor influence. These findings demonstrate that CBD protects primary hippocampal neurons from rotenone‐induced toxicity, with TRPV1 playing a central role in the mechanism.

## Graphical abstract

(–)‐Cannabidiol (CBD) protects primary hippocampal neurons from rotenone, a mitochondrial complex I inhibitor that causes cell death. CBD preserves neuronal viability and morphology, with antagonist assays showing that transient receptor potential vanilloid 1 (TRPV1) mediates this neuroprotective effect.

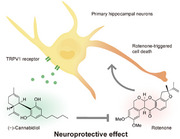

## 1 Introduction

Mitochondrial dysfunction in the brain is one of the pathophysiological commonalities associated with neurodegenerative diseases including Alzheimer's disease, Parkinson's disease (PD), Huntington's disease, and amyotrophic lateral sclerosis [1, 2, 3, 4, 5]. For example, mutations in mitochondrial DNA and increased production of reactive oxygen species (ROS) have been implicated in aging‐related neurodegenerative diseases [6, 7]. Oxytosis/ferroptosis in mitochondria, a non‐apoptotic cell‐death pathway, has been proposed to be involved in neurodegenerative diseases [8]. In this regard, a new generation of therapeutics for prevention and treatment of neurodegenerative diseases has recently been pursued with targeting mitochondrial dysfunction [9, 10, 11]. Among the natural compounds, the non‐psychoactive (–)‐cannabidiol (CBD) from the *Cannabis* genus has been reported to prevent the death of cultured HT22 neural cells in the oxygen‐glucose deprivation/reperfusion model by enhancing mitochondrial bioenergetics and modulating glucose metabolism [12, 13, 14, 15]. CBD represents a mechanistically distinct antioxidant compared with classical compounds such as vitamin E or ascorbate, as it not only scavenges reactive oxygen species but also modulates receptor‐dependent survival pathways in neurons [16, 17]. However, despite the growing evidence of CBD's neuroprotective potential, its effect on rotenone‐induced toxicity has not been closely investigated, particularly for primary neural cells. Previous studies have mainly focused on immortalized cell lines or specific neuronal subtypes, leaving a gap in understanding its action in primary hippocampal neurons.

Rotenone, a potent inhibitor of mitochondrial complex I (NADH:ubiquinone oxidoreductase) [18, 19] and also an inhibitor of microtubule assembly [20, 21], is toxic to various cell types [22, 23, 24, 25, 26, 27, 28, 29, 30], particularly dopaminergic neurons, the degeneration of which contributes to PD [31, 32, 33, 34]. Hence, rotenone has widely been used as a PD‐inducing model compound [35, 36]. As a complex‐I inhibitor, rotenone inhibits the electron transfer from the Fe‐S centers in the complex I to ubiquinone, blocking oxidative phosphorylation in the mitochondrial respiratory chain, leading to limited ATP production, ROS formation, and apoptosis [37, 38, 39]. For instance, the viability of cultured cerebrocortical neurons decreases by 30% within 1 day after exposure to rotenone (1 µM) [40]. Rotenone also has been reported to be toxic to hippocampal neurons, inhibiting axonogenesis at sub‐apoptotic concentrations (0.1 µM) [41, 42], although the neurotoxicity profiles of rotenone in higher concentrations (>0.1 µM) have not been investigated in the studies. Considering previous reports, rotenone serves as a suitable defect‐inducer for investigating the neuroprotective effects of a compound in a cell‐culture setting.

Some natural compounds have been reported to show neuroprotective effects against rotenone‐induced toxicity, albeit few reports for primary neural cells [43, 44]. For example, a recent study has shown that CBD and cannabigerol (CBG) noticeably attenuates cell death, induced by rotenone (40 nM), in the in vitro culture of primary cerebellar granule neurons [45]. CBD also ameliorates the neurotoxicity of rotenone on tyrosine‐hydroxylase‐specific, dopaminergic neurons in the mesencephalic culture [46]. On the other hand, Epifractan (EPI), a pharmaceutical extract of *Cannabis* with a high CBD content, has demonstrated strong neuroprotection against rotenone‐induced toxicity in primary cerebellar granule neurons. EPI showed effects comparable to those of XALEX, a purified CBD formulation, reinforcing CBD as a key active component in neuroprotection. Additionally, formulation factors, such as medium‐chain triglyceride oil, have been suggested to influence bioavailability of CBD and enhance its protective properties [47]. In a related study, cannabinol (CBN), a minor phytocannabinoid from *Cannabis*, has been reported to protect HT22 and SH‐SY5Y neuroblastoma cell lines from RSL3‐induced mitochondrial dysfunction, proposedly by inhibiting the oxytosis/ferroptosis pathway [48]. Consistently, a pre‐clinical Alzheimer's disease screening study has demonstrated that cannabinoids, including CBN, provide neuroprotective effects under similar conditions [49]. As part of our ongoing research on the effects of CBD on primary neural cells [13, 50, 51], this work investigates the neuroprotective effect of CBD against rotenone‐induced toxicity in primary hippocampal neurons. We systematically evaluated rotenone toxicity in cultured hippocampal neurons and investigated neuroprotective effects of CBD at concentrations below 10 µM, based on previous reports on CBD toxicity in neural cells [45, 50, 51].

## 2 Results and Discussion

Rotenone is a broad‐spectrum lipophilic pesticide known to cross cellular membranes and induce neurotoxicity by inhibiting mitochondrial complex I [52]. Rotenone has been extensively used as a botanical insecticide and piscicide, and its well‐characterized neurotoxic profile has made it one of the most frequently employed agents for modeling PD in animals [53, 54]. The inhibition of mitochondrial complex I disrupts electron transport, leading to increased mitochondrial ROS production, oxidative stress, and impairment of essential cellular processes [55]. To investigate the neurotoxic effects of rotenone in vitro, we assessed the susceptibility of primary hippocampal neurons to various concentrations of rotenone.

Hippocampal tissues dissected from E18 Sprague‐Dawley rat pups were dissociated and seeded onto poly‐d‐lysine (PDL)‐coated coverslips at a density of 100 cells mm^−2^. Cultures were maintained in the neuron culture medium (NB) for 24 h, after which the medium was replaced with fresh NB containing rotenone and incubated for an additional 24 h. As a control, neurons were treated with 0.1% DMSO diluted in NB. To examine the dose‐dependent effects of rotenone, neurons were treated with rotenone at a series of concentrations ranging from 1 to 500 nM (1, 10, 20, 40, 60, 100, 200, and 500 nM). Cell viability was assessed using the LIVE/DEAD viability/cytotoxicity assay, in which intracellular esterases in viable cells convert calcein AM to green fluorescent calcein (*λ*_emission_: 515 nm), while ethidium homodimer‐1 (EthD‐1) binds to nucleic acids in membrane‐compromised cells, emitting red fluorescence (*λ*_emission_: 617 nm). Viability was expressed as a percentage relative to the untreated group (%viability = viability of the sample divided by viability of the reference × 100) in this paper.

**FIGURE 1:**
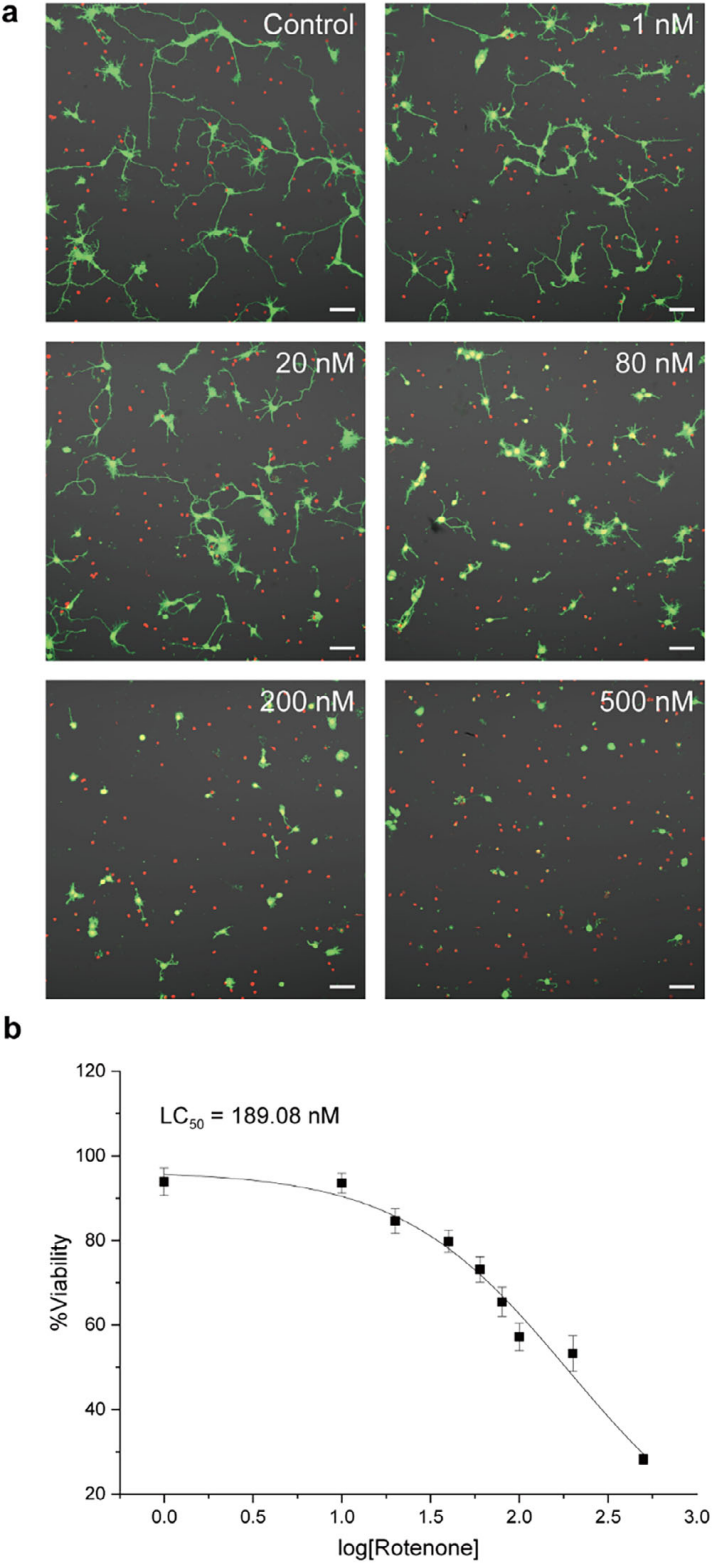
Neurotoxic effects of rotenone on primary hippocampal neurons. Primary hippocampal neurons were treated with rotenone, which was diluted to final concentrations of 1–500 nM (1, 10, 20, 40, 60, 100, 200, and 500 nM) in the NB. (a) Representative CLSM images of neurons after live/dead staining. Green: calcein AM (live), Red: EthD‐1 (dead). Scale bar: 50 µm. (b) Dose–response curve showing %viability (*y*‐axis) as a function of log[Rotenone] (*x*‐axis). LC_50_ was determined as the concentration at which %viability decreased to 50%. Data are presented as mean ± S.E. (*n* = 3). S.E. indicates standard error.

Figure 1 illustrates the neurotoxic effects of rotenone on cultured primary hippocampal neurons. Confocal laser‐scanning microscopy (CLSM) analysis revealed a concentration‐dependent reduction in neurite outgrowth, accompanied by decreased viability (Figure 1a, green: live, red: dead). For example, at the highest concentration of rotenone tested (500 nM), most neurons displayed a rounded morphology with only the soma remaining, indicative of severe neurite degeneration. The %viability was observed to be 28.2% for 500 nM of rotenone. Based on the viability data, the lethal concentration 50 (LC_50_)—the rotenone concentration at which the %viability is reduced to 50%—was calculated as 189.1 nM (Figure 1b). For subsequent neuroprotection studies, 200 nM of rotenone was selected as the reference condition.

Our previous study demonstrated that CBD at concentrations below 10 µM exhibited minimal toxicity in neuronal cells [50]. Accordingly, we selected 2.5 µM CBD to investigate its effects on rotenone‐induced neurotoxicity. At this concentration, neuronal viability was comparable to that of the vehicle control (0.1% DMSO), with no statistically significant difference between the two groups (100.5% vs. 99.8%) (Figures S1 and S2). To evaluate the neuroprotective ability of CBD against rotenone‐induced toxicity, neurons were treated with 200 nM rotenone in the presence of 2.5 µM CBD. Neurons were seeded onto PDL‐coated coverslips at a density of 100 cells mm^−2^ and incubated in NB for 1 day in vitro (DIV) for stabilization. After incubation, neurons were treated with CBD and rotenone, where CBD was either co‐treated with rotenone or pre‐treated 1 h before rotenone treatment.

**FIGURE 2:**
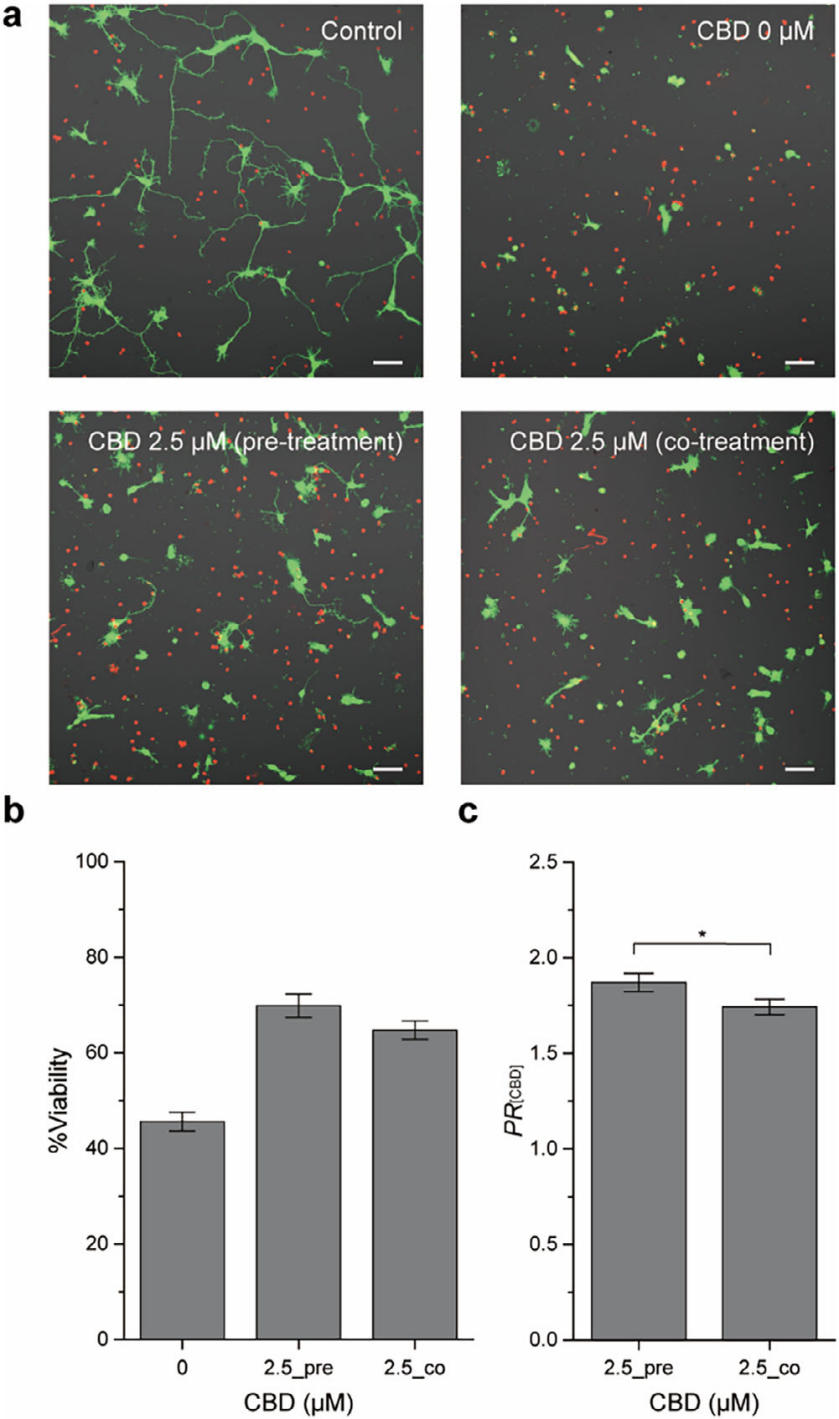
Neuroprotective effect of CBD against rotenone‐induced toxicity in primary hippocampal neurons. Neurons were treated with 200 nM rotenone alone, or with 200 nM rotenone and 2.5 µM CBD. CBD was either co‐treated with rotenone or pre‐treated 1 h prior to rotenone exposure. (a) Representative CLSM images of neurons. Green: calcein AM (live), Red: EthD‐1 (dead). Scale bar: 50 µm. (b) Quantification of %viability for each group. (c) Protection ratio (*PR*_[CBD]_), calculated as the ratio of %viability in CBD‐treated groups to that in the rotenone‐only group. All groups were treated with the same concentration of rotenone (200 nM). Data are presented as mean ± S.E. (*n* = 11). *p < 0.05.

Treatment with 200 nM rotenone alone resulted in a decreased neuronal viability of 45.6%, with CLSM images revealing pronounced neurite shrinkage compared with the vehicle control (Figure 2a). In stark contrast, the CBD pre‐treatment (2.5_pre) elevated %viability to 69.9%, while the CBD co‐treatment (2.5_co) resulted in 64.7% viability (Figure 2b). Both treatment conditions showed statistically significant improvements in viability compared with the rotenone‐only group. This outcome contrasts with our previous findings in primary hippocampal neurons exposed to hydrogen peroxide (H_2_O_2_) [50], where a 1‐h pre‐treatment of CBD (5 µM) did not improve cell viability, while the CBD co‐treatment attenuated H_2_O_2_‐induced cytotoxicity. In contrast, a CBD pre‐treatment in primary cerebellar granule neurons has been reported to confer protection against rotenone‐induced neurotoxicity [45, 47]. In the mesencephalic culture, the CBD co‐treatment with rotenone elevated dopaminergic neuronal survival from 29% under rotenone alone to 42%, although this remained below the ∼80% viability observed with CBD alone, indicating only partial protection [46]. Taken together, these results suggest that although the precise outcomes may vary with the nature of the toxic stimulus and experimental conditions, CBD consistently exerts neuroprotective effects in primary neurons. Importantly, the present study demonstrates that against rotenone toxicity, CBD provides marked protection under both pre‐ and co‐treatment conditions.

To quantify the protective effect of CBD, we calculated the protection ratio of CBD (*PR*_[CBD]_) as the %viability of CBD/rotenone‐treated neurons divided by that of the rotenone‐only group. The *PR*_[CBD]_s for the CBD pre‐treatment (2.5_pre) and co‐treatment (2.5_co) groups were 1.87 and 1.74, respectively, showing a statistically significant difference between the two treatment groups (*p* < 0.05) (Figure 2c). At a higher concentration of 5 µM, CBD exhibited a lower protection ratio than 2.5 µM (Figure S3).

**FIGURE 3:**
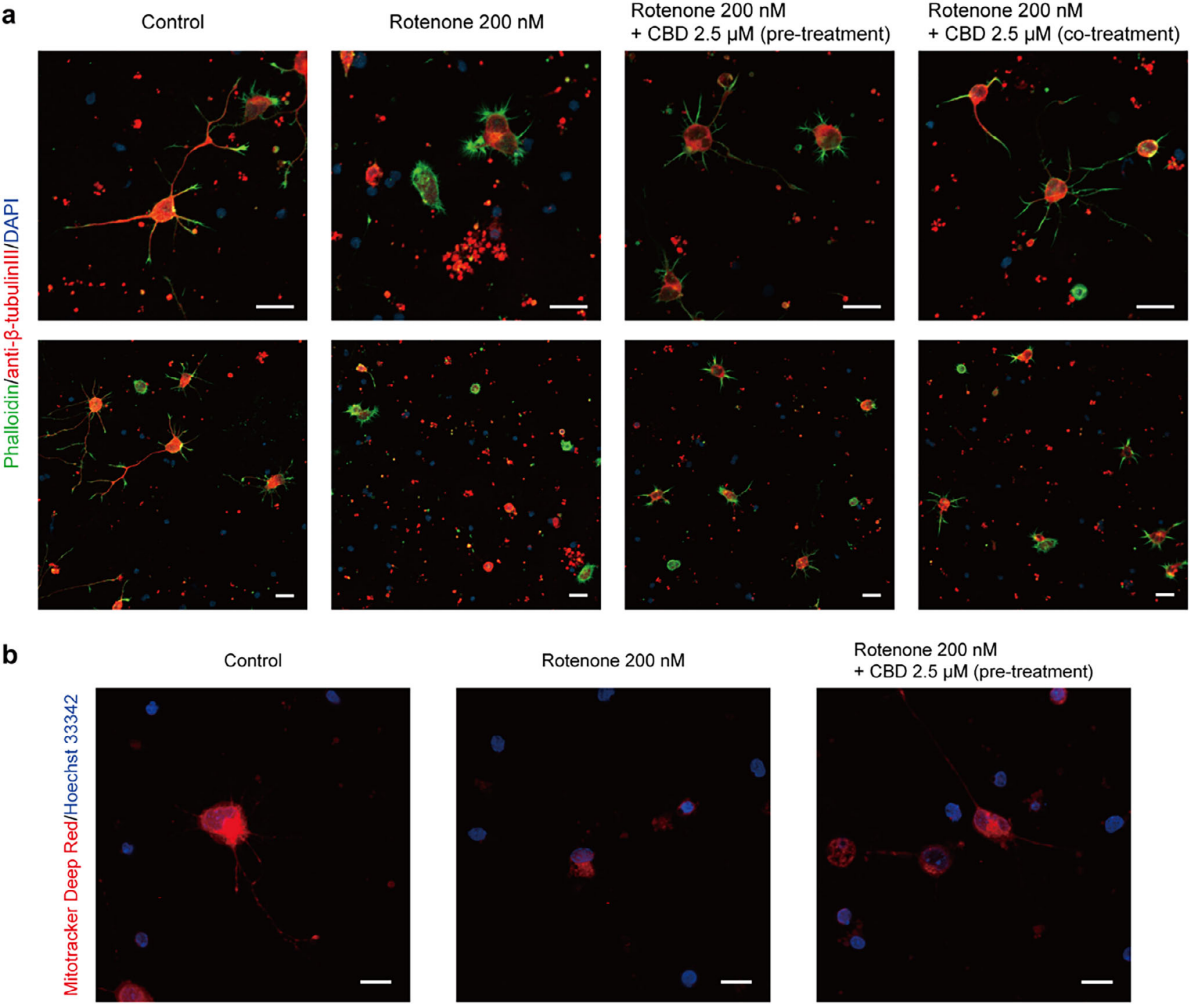
CLSM images of hippocampal neurons treated with rotenone and CBD. (a) Neurons were treated with 200 nM rotenone alone, or with 200 nM rotenone and 2.5 µM CBD, either co‐treated or pre‐treated 1 h prior to rotenone exposure. Cells were stained for F‐actin (green), *β*‐tubulin III (red), and nuclei (blue). Scale bar: 20 µm. (b) Neurons were stained with MitoTracker Deep Red after treatment with rotenone and CBD. Neurons were treated with 200 nM rotenone alone or with 200 nM rotenone and 2.5 µM CBD (1‐h pre‐treatment). Cells were stained with MitoTracker Deep Red (red: mitochondria) and Hoechst 33342 (blue: nuclei). Scale bar: 10 µm.

To further investigate the neuroprotective effects of CBD, immunocytochemistry was performed to evaluate neuronal morphology. *β*‐Tubulin III and F‐actin were immunolabeled with an anti‐*β*‐tubulin III antibody and phalloidin, respectively, and visualized by CLSM (Figure 3a). Control neurons (without rotenone treatment) exhibited well‐developed neurite outgrowth with long and extended dendrites. In contrast, the neurons exposed to 200 nM rotenone showed a marked reduction in axonal branching, with the majority of cells displaying only nuclei or signs of membrane rupture and fragmentation. Remarkably, both pre‐treatment and co‐treatment with 2.5 µM CBD preserved axonal branching and neurite structures to a greater extent despite rotenone exposure. Although a small number of ruptured cells were observed, overall neurite morphology was markedly improved compared with the rotenone‐only group. To further quantify these morphological differences, neurons were categorized into three morphological types—morph‐1, morph‐2, and morph‐3—corresponding to the neuronal morphologies characteristic of developmental stages (stage‐1, stage‐2, and stage‐3 or more advanced) described by Dotti et al. [56]. Morph‐3 neurons were predominant (77%), followed by morph‐2 (19%) and morph‐1 (4%) in the control group (Figure S4). In contrast, rotenone treatment (200 nM) markedly decreased the proportion of morph‐3 neurons (15%), while increasing morph‐1 (57%) and morph‐2 (28%) populations. Co‐treatment with CBD (2.5 µM) shifted the neuronal distribution towards higher morphological stages (morph‐1: 30%, morph‐2: 32%, morph‐3: 32%), and a similar trend was observed under the 1‐h pre‐treatment condition (morph‐1: 31%, morph‐2: 41%, morph‐3: 27%).

To gain insight into the protective effects of CBD, we assessed mitochondrial activity using MitoTracker Deep Red (*λ*_emission_: 665 nm), a membrane potential‐sensitive dye that preferentially accumulates in active mitochondria. Since MitoTracker accumulation depends on the inner mitochondrial membrane potential, it enables visualization of active mitochondria [57]. At low concentrations (≤ 100 nM), MitoTracker Deep Red has minimal impact on complex I activity, providing an effective marker of mitochondrial function [58]. Therefore, neurons were stained with MitoTracker Deep Red at a concentration of 100 nM, and live‐cell imaging was performed using CLSM (Figure 3b). In untreated neurons, strong red fluorescence was clearly observed, reflecting intact and active mitochondria. In contrast, neurons treated with 200 nM rotenone exhibited reduced MitoTracker Deep Red fluorescence in the cytosol, and mitochondria appeared unevenly distributed, with signs of fragmentation or aggregation rather than uniform cytosolic dispersion. When neurons were pre‐treated with 2.5 µM CBD 1 h prior to rotenone exposure, overall mitochondrial fluorescence intensity was higher compared with the rotenone‐only group, although some cells still showed reduced fluorescence and fragmentation (Figure S5). Collectively, these results showed the protective effect of CBD in preserving mitochondrial activity.

CBD has been reported to interact with various molecular targets in neurons, but only a subset appears to be directly involved in the regulation of neuronal survival under mitochondrial stress. Among these, serotonin 1A receptor (5‐HT_1A_R) and transient receptor potential vanilloid 1 (TRPV1) have been highlighted for their roles in modulating calcium homeostasis, neuronal excitability, and apoptotic signaling [13, 14]. Based on these findings, we selected 5‐HT_1A_R and TRPV1 to investigate how CBD influences neuronal responses in the context of rotenone‐induced neurotoxicity.

5‐HT_1A_R is a Gi/o‐coupled receptor that contributes to neuronal survival by decreasing membrane excitability and activating pro‐survival intracellular signaling pathways [13]. While CBD has been reported to enhance these protective effects, whether this modulation occurs through direct receptor binding or downstream signaling remains unclear. In contrast, TRPV1 regulates calcium influx and participates in both sensory and inflammatory signaling under physiological conditions [59]. However, sustained or excessive activation of TRPV1 can disrupt calcium balance and mitochondrial homeostasis, leading to oxidative stress and apoptosis. CBD modulates TRPV1 activity in a context‐dependent manner, suggesting that it may influence neuronal outcomes differently depending on the cellular environment. Given that rotenone‐induced neuronal toxicity involves mitochondrial dysfunction and oxidative stress [39], both TRPV1 and 5‐HT_1A_R could potentially modulate neuronal vulnerability under such stress. For example, TRPV1 may amplify stress responses by promoting calcium overload and ROS generation, while 5‐HT_1A_R activation could counteract these effects through anti‐apoptotic pathways.

**FIGURE 4:**
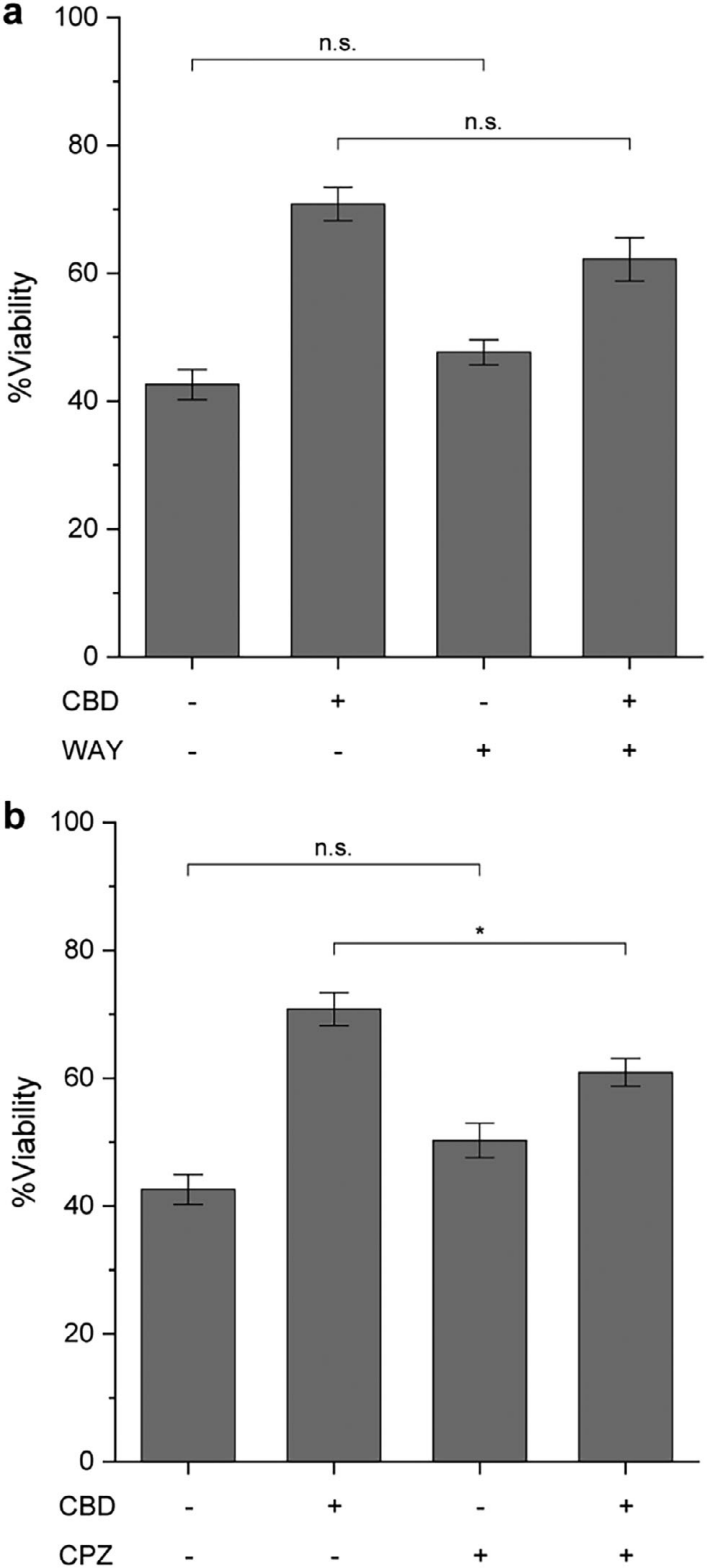
A graph of %viability after 24 h treatment of primary hippocampal neurons (1 DIV) with CBD and antagonists in the presence of rotenone (200 nM). (a) Neurons treated with the 5‐HT_1A_R antagonist WAY (10 µM). (b) Neurons treated with the TRPV1 antagonist CPZ (10 µM). CBD (2.5 µM) was co‐treated in each condition. WAY: (*S*)‐WAY100135, CPZ: capsazepine. Data are presented as mean ± S.E. (*n* = 30). *p < 0.05, n.s. indicates not significant.

To investigate the contribution of TRPV1 and 5‐HT_1A_R to the neuroprotective mechanism of CBD in our studies, primary hippocampal neurons were pre‐treated with CBD (2.5 µM) for 1 h, followed by rotenone (200 nM) treatment together with either the TRPV1 antagonist capsazepine (CPZ) or the 5‐HT_1A_R antagonist (*S*)‐WAY100135 (WAY). As references, neuronal viability increased from 42.6% in the rotenone‐only group to 70.8% with CBD treatment (Figure 4). As a control to account for possible off‐target effects of the antagonists, neurons were co‐treated with each antagonist and rotenone in the absence of CBD. Under these conditions, cell viability was comparable to that of the rotenone‐only group, indicating that the antagonists themselves did not influence rotenone‐induced toxicity. In contrast, co‐treatment of CBD with either CPZ or WAY partially reduced the protective effect, lowering neuronal viability to 60.9% and 62.2%, respectively. Among these, only the CPZ‐treated group showed a statistically significant reduction in viability compared with the CBD/rotenone group. These findings suggest that TRPV1, rather than 5‐HT_1A_R, plays a more prominent role in mediating the neuroprotective action of CBD in our rotenone‐induced neuronal toxicity model. Overall, our data are consistent with TRPV1‐dependent signaling reported in neuronal models. Although we cannot yet distinguish direct receptor activation from downstream involvement, the results support a TRPV1‐linked pathway underlying CBD's protective effect against rotenone‐induced toxicity [60, 61, 62].

Consistent with these results, previous studies using a neuron‐with‐neuron sandwich platform—where two neuron‐cultured plates were assembled face‐to‐face to promote intercellular interactions—demonstrated that the inhibition of TRPV1 with CPZ prevented the neurotoxic effects of high CBD concentrations (30 µM) [59]. Our current finding further highlights the involvement of TRPV1 in cannabinoid‐induced neuronal responses, particularly under conditions of excessive stimulation. While 5‐HT_1A_R has been implicated in the neuroprotective effects of another cannabinoid, CBG [45], our results suggest a limited role for 5‐HT_1A_R in CBD‐mediated protection under mitochondrial stress conditions. Taken together, it could be concluded that TRPV1 serves as a key mediator of CBD‐induced neuroprotection in primary hippocampal neurons, whereas 5‐HT_1A_R plays only a minor role.

## 3 Conclusion

In summary, this study demonstrates that CBD effectively protects primary hippocampal neurons from rotenone‐induced toxicity by maintaining neuronal viability and preserving neurite morphology. Both pre‐treatment and co‐treatment with CBD effectively attenuated rotenone‐induced cell death, and morphological analyses confirmed the preservation of axonal branching and neuronal structure. Consistent with our findings, several in vivo studies have reported that cannabis‐derived phytocannabinoids attenuate oxidative stress and neuronal degeneration induced by rotenone administration in animal models [63, 64]. These in vivo observations reinforce the neuroprotective potential of CBD and further support our in vitro findings at the cellular level. Confocal imaging with MitoTracker further supported these findings by showing that CBD partially maintained mitochondrial activity compared with rotenone‐treated neurons. Importantly, antagonist experiments revealed that this protective effect was markedly reduced in the presence of a TRPV1 antagonist, whereas inhibition of the 5‐HT_1A_R showed a minor influence. The modest neuroprotective effect observed in this study likely reflects intrinsic limitations of in vitro cultures, which lack astrocytic and glial support that normally sustain neuronal metabolism and redox homeostasis [13, 65]. These observations suggest that TRPV1 plays a prominent role in mediating CBD's neuroprotective action in our system, while the involvement of 5‐HT_1A_R appears limited. Taken together, these findings provide a mechanistic understanding of how CBD supports neuronal survival under rotenone‐induced stress and emphasize the significance of TRPV1 modulation as a key determinant of neuroprotection.

## Conflicts of Interest

The authors declare no conflicts of interest.

## Supporting information




**Supporting file 1**: asia70441‐sup‐0001‐SuppMat.pdf

## Data Availability

The data that support the findings of this study are available in the supplementary material of this article.
