# Supplementary material for: Neuroprotective Effect of Cannabidiol Against Rotenone in Hippocampal Neuron Culture
Source: Chem Asian J. 2025 Nov 19;20(24):e00946. doi: 10.1002/asia.202500946 (PMC12711387; doi:10.1002/asia.202500946)
Supplement: Supplementary file 1 — Supporting file 1: asia70441‐sup‐0001‐SuppMat.pdf [file ASIA-20-e00946-s001.pdf]

Supporting Information

Neuroprotective Effect of Cannabidiol Against Rotenone in Hippocampal Neuron Culture

*Seoin Yang, Hyunwoo Choi, Jungnam Kim, and Insung S. Choi**

Table of Contents

- Experimental.
- **Figure S1.** CLSM images of primary hippocampal neurons treated with CBD.
- **Figure S2.** Viability analysis of neurons with vehicle or CBD.
- **Figure S3.** Protection ratio of neurons treated with different concentration of CBD.
- **Figure S4.** Neuronal development-stage distributions after CBD treatment.
- **Figure S5.** CLSM images of neurons stained with MitoTracker Deep Red.
- **Figure S6.** Viability analysis of antagonist-treated neurons.

Experimental

Materials. CBD was purchased from Cayman Chemical Company. Rotenone (Sigma-Aldrich), phosphate-buffered saline (PBS, pH 7.4, Welgene), Trypsin-EDTA solution (1×, Welgene), penicillin-streptomycin (P/S, 5,000 U mL^{-1} of penicillin and 5,000 mg mL^{-1} of streptomycin, Welgene), Neurobasal™ Plus medium (Thermo Fisher Scientific), B-27™ plus serum-free supplement (50×, Thermo Fisher Scientific), GlutaMAX™ supplement (100×, Thermo Fisher Scientific), Hank's Balanced Salt Solution (HBSS, Welgene), calcein AM (Invitrogen™), ethidium homodimer-1 (EthD-1, Invitrogen™), poly-D-lysine (Sigma-Aldrich), dimethyl sulfoxide (Sigma-Aldrich), Alexa Fluor™ 488 phalloidin (Invitrogen™), Alexa Fluor™ 594 goat anti-rabbit secondary antibody (Invitrogen™), Hoechst 33342 trihydrochloride (1 mg mL^{-1}, Invitrogen™), paraformaldehyde (Sigma-Aldrich), Triton™ X-100 (Sigma-Aldrich), anti-*β*-tubulin III antibody produced in rabbit (Sigma-Aldrich), bovine serum albumin (BSA, Sigma-Aldrich), Mitotracker™ deep red FM (Thermo Fisher Scientific), (*S*)-WAY100135 dihydrochloride (WAY, Tocris Science), Capsazepine (CAP, Sigma-Aldrich), and antifade mounting medium with 4',6-diamidino-2-phenylindole (DAPI, Vector Laboratories) were used as received. WAY or CAP was dissolved, to a 10 mM stock, in dimethyl sulfoxide (DMSO, Junsei) and stored at -20 °C. Deionized water (DI water, 18.2 MΩ·cm) from a Milli-Q Direct 8 (Millipore) was used. E18 Sprague-Dawley rats were obtained from KOATECH. This study was approved by IACUC (Institutional Animal Care and Use Committee) of KAIST (KA2025-064-v1).

Neuron Culture. Primary hippocampal neurons were isolated from the hippocampi of E18 Sprague-Dawley rat fetuses. Dissected tissues were incubated in a trypsin-EDTA solution for 10 min and washed six times with HBSS. The hippocampi were then gently dissociated by trituration and centrifuged at 1000 rpm for 3 min. The resulting cell pellet was resuspended in neuron culture plus medium (NB^+), consisting of neuron culture medium (NB) supplemented with 12.5 μM L-glutamic acid. NB was composed of Neurobasal™ Plus Medium supplemented with 1% (v/v) P/S, 2% (v/v) B-27™ Plus Supplement and 1% (v/v) GlutaMAX™ Supplement. The cell suspension was filtered through a cell strainer and seeded onto poly-D-lysine (PDL)-coated coverslips at a density of 100 cells mm^{-2}. Neurons were then cultured at 37 °C in a humidified incubator with 5% CO_2.

Neurotoxicity and Neuroprotection Assays. For neurotoxicity experiments, the culture medium was replaced with NB containing either CBD (2.5 μM) or rotenone (1-500 nM) at 1 DIV (days in vitro). The rotenone stock solution was freshly prepared for each experiment by dissolving rotenone in DMSO at a concentration of 10 mM. Fresh NB was added to each well and diluted with a predetermined volume of the rotenone stock solution. For neuroprotection experiments, the culture medium was replaced at 1 DIV with 990 μL of fresh Neurobasal™ Plus medium containing 2.5 μM CBD, followed by the addition of 10 μL of a 200 μM rotenone stock solution. For the 0 μM CBD control group, 0.1% DMSO was used. In a separate experimental set, neurons were preincubated with 2.5 μM CBD for 1 h at 1 DIV prior to rotenone treatment. To examine receptor-specific mechanisms, antagonists were applied to investigate the involvement of 5-HT_{1A}R and TRPV1 receptors in the neuroprotective effects of cannabinoids. Specifically, (*S*)-WAY100135 (5-HT_{1A}R antagonist, 10 μM) or capsazepine (TRPV1 antagonist, 10 μM) was co-incubated with CBD (2.5 μM) and rotenone (200 nM) for 24 h.

Characterizations. *(a) Viability assay:* Cell viability was assessed using the LIVE/DEAD® Viability/Cytotoxicity Kit, which contained calcein AM and EthD-1. Cells were incubated with 1.6 μM calcein AM and 4 μM EthD-1 for 20 min at 37 °C, followed by fluorescence imaging using an LSM 800 confocal laser-scanning microscope (CLSM, Zeiss). Viability was quantified using ImageJ software and expressed as percentage viability (%viability, mean ± standard error) based on at least three independent cultures, each performed in triplicate. *(b) Immunocytochemistry:* Neurons were fixed with 4% paraformaldehyde in PBS for 15 min at room temperature, washed three times with PBS, permeabilized with 0.1% Triton X-100 in PBS for 10 min at room temperature, and again washed three times with PBS. After blocking with 6% BSA in PBS for 30 min at room temperature, the cells were incubated with a rabbit anti-β-tubulin III primary antibody (2 μg mL^{-1}) diluted in 1.5% BSA solution for 1 h at 37 °C. Following three washes with PBS, neurons were incubated for 1 h at 37 °C with Alexa Fluor™ 488 phalloidin (0.4 μM) and Alexa Fluor™ 594 goat anti-rabbit secondary antibody (4 μg mL^{-1}), both diluted in 1.5% BSA, to visualize F-actin and β-tubulin III, respectively. After a final PBS wash, the neurons were mounted using a DAPI-containing mounting solution on coverslips and incubated for 30 min at room temperature prior to CLSM imaging. *(c) Mitochondrial staining:* At 2 DIV, the culture medium was removed and replaced with NB containing Hoechst 33342 (0.1 μg mL^{-1}) and MitoTracker™ Deep Red (100 nM). Cells were incubated at 37 °C for 30 min. After incubation, the coverslips were transferred to a confocal dish containing fresh NB, and fluorescence images were acquired using CLSM.

Statistical Analysis. Data were presented as mean ± standard error (S.E.) with sample size (n = 3-11) unless otherwise indicated. Two-group comparisons were analyzed using Student's t-test. Statistical significance was assessed at $\alpha = 0.05$ ($*p < 0.05$; n.s., not significant). Statistical analysis and graph generation were performed using OriginPro 2019.

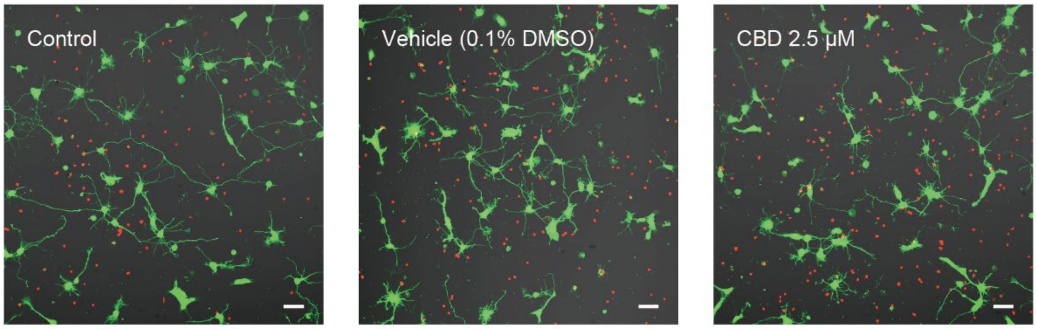

Figure S1. CLSM images of naïve neurons and neurons treated with vehicle or 2.5 μM CBD for 24 h. Green: calcein AM (live); Red: EthD-1 (dead). Scale bar: 50 μm.

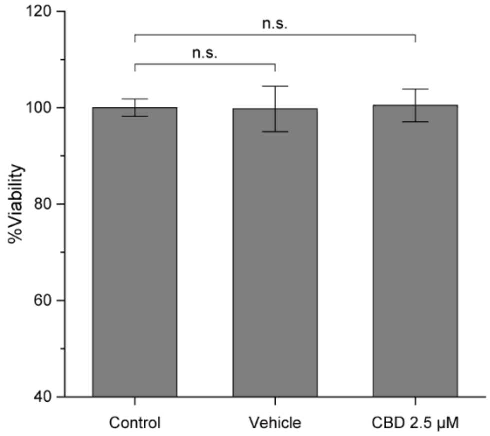

Figure S2. Neurons were treated with vehicle or 2.5 μM CBD for 24 h, and cell viability was assessed. No significant differences were observed among the control, vehicle-treated, and CBD-treated groups. Data are presented as mean ± S.E. (n = 8). n.s., not significant.

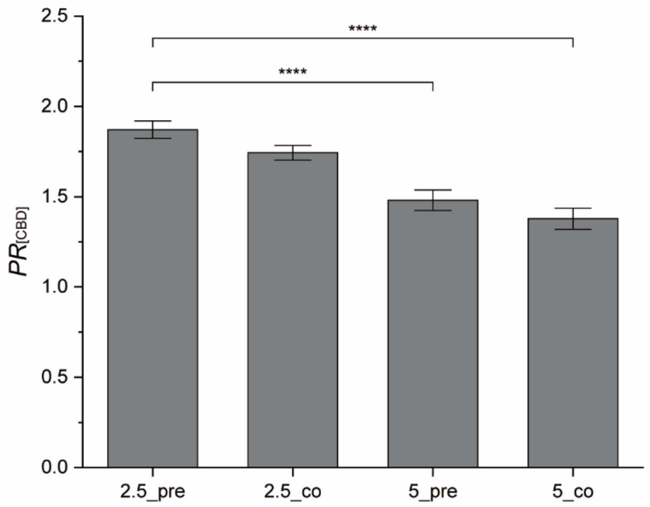

Figure S3. Protection ratio ($PR_{[CBD]}$), calculated as the ratio of %viability in 2.5 μM or 5 μM CBD-treated groups to that in the rotenone-only group. All groups were treated with the same concentration of rotenone (200 nM). Data are presented as mean ± S.E. (n = 11). *p < 0.0001.

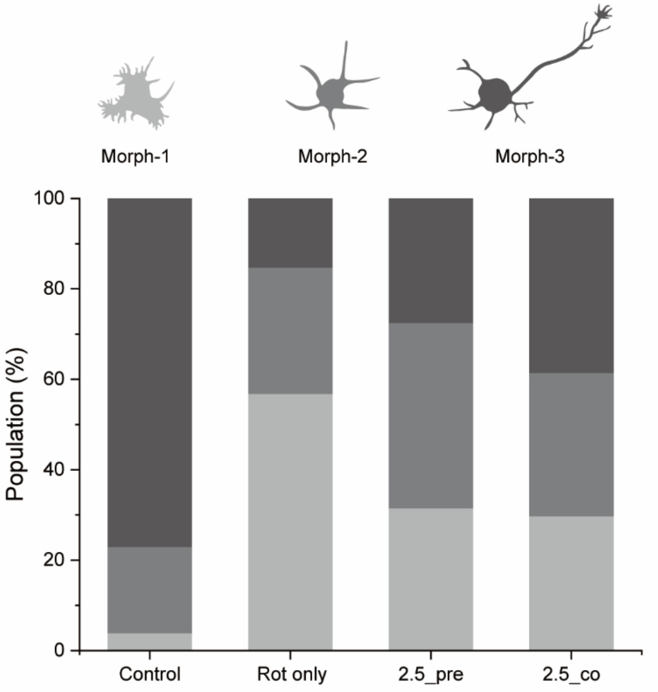

Figure S4. A graph of morphological distributions after CBD treatment. Rot only: samples treated with rotenone.

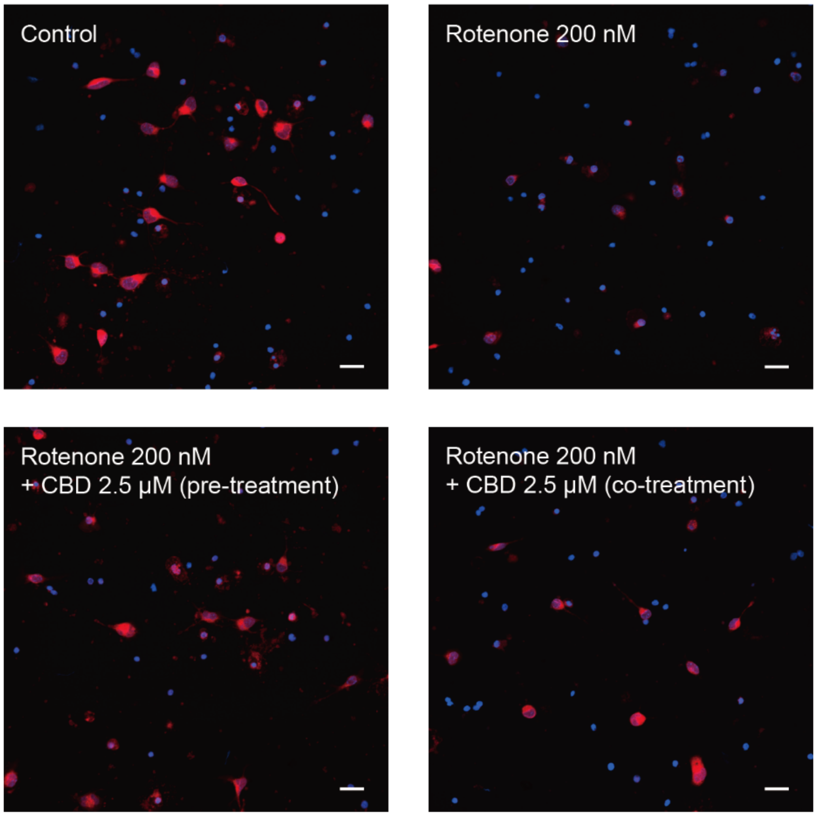

Figure S5. Low-magnification CLSM images of neurons stained with MitoTracker Deep Red and Hoechst 33342 to assess mitochondrial activity across the cell population. Red: mitochondria (MitoTracker™ Deep Red); Blue: nuclei (Hoechst 33342). Scale bar: 20 μm.

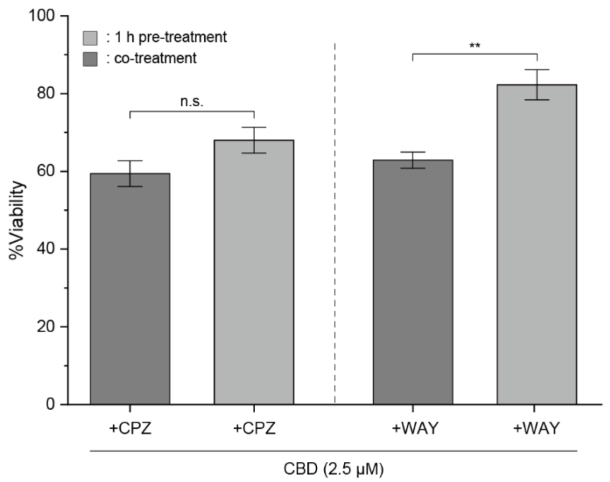

Figure S6. Neurons were treated with 200 nM rotenone together with CPZ (10 μM) or WAY (10 μM), and CBD (2.5 μM) was either co-treated or pre-treated 1 h prior to rotenone treatment. Cell viability was then assessed after 24 h. No significant differences were observed between the groups treated with CPZ, whereas the WAY-treated groups showed a significant difference between CBD co-treatment and pre-treatment. Data are presented as mean ± S.E. ($n = 6$). $**p < 0.01$; n.s., not significant.
